# Examining global biodiversity accounts: Implications of aggregating characterization factors from elementary flows in multi‐regional input–output analysis

**DOI:** 10.1111/jiec.13556

**Published:** 2024-10-08

**Authors:** Killian Davin, Maximilian Koslowski, Martin Dorber, Edgar Hertwich

**Affiliations:** ^1^ Industrial Ecology Programme, Department of Energy and Process Engineering Norwegian University of Science and Technology Trondheim Norway

**Keywords:** biodiversity footprints, environmentally extended input–output analysis, characterization factors, life cycle impact assessment, land use, industrial ecology

## Abstract

Extending multi‐regional input–output (MRIO) models with spatially explicit life cycle impact assessment (LCIA) models allows practitioners to quantify biodiversity impacts at every step of global supply chains. Inconsistencies may be introduced, however, when high‐resolution characterization factors (CFs) are aggregated so as to match the low spatial granularity of MRIO models. These aggregation errors are greater when CFs are aggregated via proxies, such as ecoregion land shares, instead of based on spatially explicit elementary stressor flows. Here, we describe our approach to tailoring application‐specific CFs for use in MRIO studies. We apply a global agricultural production model, Spatial Production Allocation Model (MapSPAM), with the LCIA database, LC‐IMPACT, to create crop‐specific national CFs. We investigated i) if the differing aggregation approaches and the increased spatial explicitness of the constructed CFs deviate substantially from those in LC‐IMPACT, and ii) what the resulting consequences for national production and consumption‐based biodiversity footprints are when combining the tailor‐made CFs with the EXIOBASE MRIO model. For the year 2020, we observe an increase in global production‐based biodiversity impacts of 23.5% for land use when employing crop‐specific CFs.

## 1 INTRODUCTION

Life cycle assessment (LCA) is a prominent tool for the assessment of the impacts of human pressures on ecosystem quality. LCA is grounded at the product level, quantifying the environmental impacts of an individual product or a service. For global studies, environmentally extended multi‐regional input‐output analysis (EE‐MRIO) can be the preferred approach, reducing inventory requirements and avoiding supply chain truncation (Majeau‐Bettez et al., 2011; Wiedmann et al., 2011). Both approaches translate mapped environmental pressures to ecosystem impacts via the same life cycle impact assessment (LCIA) methods.

To tackle the fact that ecosystems are heterogeneous and respond differently to environmental stress at different locations, regionalized LCIA methods, like LC‐IMPACT (Verones et al., 2020), were constructed with spatially explicit characterization factors (CFs) for several impact pathways at the relevant scales of consequence. LCA practitioners must reconcile spatial differences between life cycle inventory (LCI) datasets and the regionalized biodiversity CFs. This practice generally requires the aggregation of the more granular CFs to the spatial resolution of the LCI data. Outside the field of ecology and those individual LCA practitioners with expert ecological knowledge, the limitations of these CF aggregation approaches are poorly understood among LCA and IO experts. National CFs are used without fully acknowledging the uncertainty that arises when CFs, created to measure species impacts across distinct ecological zones, are condensed into singular values to represent biodiversity impacts at the country level.

Our aim is to contribute toward the understanding of how differing native scale aggregation approaches affect the results of biodiversity impact assessments in LCA and environmentally extended input‐output analysis (EEIO). Additionally, we would like to revisit our understanding of the global consequences of consumption and production on biodiversity. In this paper, we focus on the sensitivity of national and continental CFs for agricultural land use in LC‐IMPACT and analyze the impacts on biodiversity footprints through the use of EEIO modeling.

Lenzen et al. (2012) were the first to model potential ecosystem quality damages with EE‐MRIO models, mapping the MRIO database, Eora (Lenzen et al., 2013), with the Red List of Threatened Species by the International Union for Conversation of Nature (IUCN) to assess the impacts of global trade. Lenzen circumvented the absence of models estimating the causal relationship between environmental pressure and regional ecosystem impact, and relied instead on threat causes provided in the red lists, such as “smallholder farming” and “logging and wood harvesting.” These causes were then connected in the input–output tables to specific industries, such as farming and forestry. Moran and Kanemoto (2017) followed, linking consumption to spatially resolved IUCN species threat hotspots, concluding that the scale of biodiversity impacts are dependent upon local biodiversity rates and their relative sensitivity to human pressures.

There was a need for a more mechanistic approach to ecosystem assessment where causal links between a unit of environmental pressure and ecosystem impacts could be made. From this need sprung LC‐IMPACT (Verones et al., 2020). The methodology measured ecosystem impacts by assessing the “potentially disappeared fraction” (PDF) of local species as a result of a unit of environmental pressure for several different impact pathways. Verones et al. (2017) and Koslowski et al. (2020) applied CFs from LC‐IMPACT with MRIO databases, to analyze the ecosystem consequences of resource footprints and the connection between urbanity and affluence for interpreting levels of consumption‐based biodiversity impacts.

EE‐MRIO databases today are not of the required spatial detail to capture the variable nature of biodiversity impacts (Crenna et al., 2020; Winter et al., 2017). Ecologists have compiled data on the relationship between species and their respective ecoregion land area via a model called the countryside species area relationship (c‐SAR). Considering the differences in ecoregions and species' responses to land occupation is important in understanding ecosystem damage. Until recently, data on land and water use were available at the country‐level only. When CFs are combined into a universal characterization at the national level, this information is distorted. This contradicts the idea that ecosystem damage is heterogeneous and overlooks the spatial variations in crop cultivation. This can lead to additional uncertainty when using LCIA regionalized methods (Moran et al., 2016; Moran & Kanemoto, 2017; Verones et al., 2017).

The largest source of uncertainty in biodiversity impact footprints likely originates from the aggregated CFs (Koslowski et al., 2020). Mutel et al. (2019) highlighted that method developers have also handled aggregation in a patched and disjointed manner. While methods are able to characterize the average flow correctly, there is no information on the size of the potential error when applied to specific elementary flows.

This paper will examine the impact of different approaches to aggregating land use characterization factors (CFs) in the context of global agricultural land use. In LC‐IMPACT, CFs are aggregated using weighted averages of national ecoregion land shares. We will compare this approach with an alternative method using land use statistics from the Spatial Production Allocation Model (MapSPAM) by the International Food Policy Research Institute (2024). MapSPAM provides local crop production data for 46 crops on a global scale, which allows for an aggregation approach based on elementary flows as recommended by Mutel et al. (2019).

## 2 METHODS

### 2.1 Overview

In the following, we outline our approach to calculating the biodiversity footprints of land use due to global agriculture and food production in the year 2020; we performed similar calculations for water use, for which we disclose the details in the SI. We employed the database EXIOBASE 3 (Stadler et al., 2018) for the MRIO analysis, providing economic data and stressor detail for land use. The MRIO pressure footprint results are then characterized to final endpoint biodiversity impacts. Required for characterization, we computed spatially explicit, crop specific, national and continental CFs based on LC‐IMPACT base data (Verones et al., 2020) and the MapSPAM model by International Food Policy Research Institute (2024).

### 2.2 MRIO database: EXIOBASE

EE‐MRIO integrates environmental factors into conventional MRIO models, tracking environmental burdens associated with the trade of goods and services (Ivanova et al., 2017; Lenzen et al., 2012; Peters & Hertwich, 2004; Verones et al., 2017). In this study, we employed the EE‐MRIO model, EXIOBASE (Stadler et al., 2018). The model was constructed with the specific aim of providing a high level of detail for environmental stressors and a consistent level of sector detail. It has a superior level of sector detail and superior number of environmental extensions when compared to competing MRIO databases like Eora (Lenzen et al., 2013) and the Global Trade Analysis Project (GTAP) (Corong et al., 2017). EXIOBASE lacks the level of spatial detail that Eora provides with only 49 regions accommodated to Eora's 189. However, a more comprehensive environmental satellite account and a homogeneous sector representation recommend its use for our case, as our procedure relies on sector mappings between models. A new generation of MRIO models, like GLORIA (Lenzen et al., 2022, 2017) and EMERGING (Huo et al., 2022), have been established with near real‐time time‐series data, expanded country resolution, and similar product detail to that of EXIOBASE. Unfortunately, these models were not available for analysis when the work on the current study commenced.

We apply version 3.8.2 of EXIOBASE and the 200×200 (product×product) monetary tables for the year 2020 (Stadler et al., 2021). We use the product×product specification, because we are interested in the biodiversity impacts associated with agricultural goods; later, we map the EXIOBASE products to those detailed in MapSPAM. The agriculture stressors related to crop production in the domains of land use and blue water consumption are included here from the environmental satellite account. The resulting stressor matrix comprises of 8 land use and 13 blue water consumption stressors. No distinction is made between final demand categories and all seven EXIOBASE final demand categories are included in the analysis of ecosystem consequences driven by final consumption of primary agriculture and food products. The product×product tables in EXIOBASE detail 8 primary crop products, 7 animal products, and 12 processed food products, including beverages. We model the crop‐related environmental impacts driven by these 27 product categories. EXIOBASE provides IO data for 44 countries, with a particular Eurocentric focus, including the EU‐27 member states plus the United Kingdom, Norway, Croatia, and Switzerland. The 44 countries combine to account for 90% of the worlds GDP for the year 2014 (Stadler et al., 2018). The remaining 10% of the global economy is aggregated into five rest of the world (ROW) regions of Africa, the Americas, Middle East, Asia, and Europe.

### 2.3 Leontief analytical calculus

The standardized methodology for IO analysis is that of the Leontief inverse calculus (Leontief, 1936, 1970). Production‐based environmental accounts are simply equal to the factors of production matrix **F**. The consumption‐based environmental footprint is typically calculated as the combination of the environmental footprint in terms of the final demand vector, y, plus the direct pressures due to the final consumption across all final demand categories. S is the environmental stressor matrix, and L is the Leontief inverse matrix. For simplicity, we analyze here impacts purely related to the production of agriculture to service the final demand in the global economy and negate the direct impacts from final demand (Equation 1). See the SI for a detailed derivation of the Leontief calculus.

(1) E=SLy

### 2.4 Life cycle impact assessment database: LC‐IMPACT

LCIA translates emission and resource inventories to impact endpoints based on the study objectives or their relative importance (ISO, 2006). Biodiversity multipliers are sourced from an LCIA database and there are several methods available with differing impact categories and value choices (Curran et al., 2011; Mutel et al., 2019). Databases covering ecosystem damage include ReCiPe (Huijbregts et al., 2017) and IMPACT World+ (Bulle et al., 2019). LC‐IMPACT version 1.3 (Verones, 2021; Verones et al., 2020) is selected here for its granular spatial assessment of ecosystem impacts (Verones et al., 2017). LC‐IMPACT measures biodiversity impacts in PDF units per m^2^ of land use for the taxa considered. It is a measure of species richness and a proxy for ecosystem health/quality. The method includes weighted species vulnerability scores used to translate local species loss to global endpoint scores.

Land occupation impacts are only considered here, with the impacts from land transformations ignored. Land CFs are constructed at the ecoregion level using the c‐SAR model (Chaudhary et al., 2015). A mathematical model, c‐SAR describes the relationship between the area of a habitat and the number of species found within that habitat. Species affinity to different habitats are modeled which helps inform potential species loss when changes to existing habitats occur. LC‐IMPACT provides stressor characterization for three agricultural land use types: “Annual,” “Permanent,” and “Pasture” cropland (Verones et al., 2020).

### 2.5 Spatial production allocation model

In the absence of spatial detail on crop production, LC‐IMPACT calculates aggregated regional multipliers for land occupation from weighted averages based on ecoregion land shares within a region's boundaries. Regionalization and crop distinction was pursued by integrating geospatial crop production data from the MapSPAM model. MapSPAM provides georeferenced crop statistics (e.g., harvested area, production quantity, and yield) for 46 agricultural crops globally, disaggregated by different farming systems and allocated into spatially gridded units at the 5 arc min resolution (International Food Policy Research Institute, 2024). To date, the model has been published for the years 2010 and 2020. The spatial scale for LC‐IMPACT's land use CFs (the ecoregion) allows for the tailoring of CFs when the similarly scaled elementary flow data from the MapSPAM model is applied for upward aggregation. The model is applied directly with LC‐IMPACT for constructing land use CFs based on specific crop location and physical land areas for the year 2020.

### 2.6 Constructing CFs from specific crop locations and elementary flows

#### 2.6.1 Overlaying spatial data layers

Discussions from LCA experts on regionalization of LCIAs concluded that regionalized LCAs should be comparable, reproducible, and transparent (Frischknecht et al., 2019). For the regionalization and subsequent aggregation of CFs, the standardized methodology proposed by Pfister et al. (2020) for overlaying country, watershed, and ecoregion data for LCIA was followed for comparability. In our case, three spatial layers, rather than the recommended six spatial layers were used due to the nature and scope of this study. The spatial layers are: (1) the political layer (Natural Earth, 2019), desired for the application of the global MRIO study; (2) terrestrial ecoregion layer, required for the analysis of biodiversity‐related land impacts (Jolliet et al., 2018; Olson et al., 2001); (3) global watersheds layer, the recommended spatial scale for analyzing regional water stress impacts (covered in the SI) (Boulay et al., 2015; Müller Schmied et al., 2014; Pfister & Bayer, 2018, 2014).

The resulting shapefile was used to map the global crop production statistics for the year 2020 from MapSPAM on a 5 arc min spatial resolution (International Food Policy Research Institute, 2024). Mapping of water consumption statistics for agricultural crops from Pfister and Bayer (2018) was carried out at the watershed level and included in the SI. CFs from LC‐IMPACT (Verones, 2021) were overlayed at their respective native scales. The resulting polygons matched the area extents of the native scale boundaries but with internal crop statistics and native CFs included.

#### 2.6.2 CF construction

We construct an individual CF for each EXIOBASE crop stressor category (paddy rice, wheat, cereal grains not elsewhere classified (nec), sugar, oil seeds, plant‐based fibers, crops nec, and “vegetable, fruit, nuts”). From LC‐IMPACT, two of the land use CFs for cropland (“Annual” and “Permanent”) and the single CF for water stress are used at their native scale and aggregated to individual Exiobase crop CFs at the national level (Verones, 2021; Verones et al., 2020). The correspondence between LC‐IMPACT and EXIOBASE crop categories is as follows: Permanent crops ‐ vegetables, fruit, nuts; and Annual crops ‐ paddy rice, wheat, cereal grains nec, sugar, oil seeds, plant‐based fibers, and crops nec. Pasture cropland is excluded from the analysis due to the absence of production data in MapSPAM. The 46 MapSPAM crop categories are mapped to the 8 EXIOBASE crop categories. The concordance is available in the spreadsheet SI.

The crop CFs are constructed by aggregating the native regional CFs to the national and continental scales using weighted averages based on annual elementary flow quantities in each native scale domain as recommended by Mutel et al. (2019). The elementary flows are the resulting spatial footprints from MapSPAM after extending to the EXIOBASE crop categories. Aggregated land CF calculation for the eight disaggregated annual crop and permanent crop land use types were as described in Equation (2), with variables as per Table 1.

(2) $$LC_{jx}=\sum_{i=1}^{p}\frac{P_{ijx}}{\sum_{i=1}^{p}P_{ijx}}\times C_{ix}$$

**TABLE 1:** Variables required in Equation (2) for the aggregation of native scale land use characterization factors to the national level.

Variable name	Description	Unit
$C_{ix}$	LC‐IMPACT land use CF for polygon *i*	$\text{PDF}\;/{\text{km}}^{2}$
$LC_{jx}$	Land use CF for crop j in country/ROW region *x*	$\text{PDF}/{\text{km}}^{2}$
$P_{ijx}$	Physical production area of crop category *j* in polygon *i*	${\text{km}}^{2}$
p	Number of polygons in country/ROW region *x*	

#### 2.6.3 Treatment of missing data

Missing CFs at the ecoregion level arising from the lack of data for isolated areas or for areas where it is reasonable not to have a CF are treated via differing techniques depending on the circumstance of the no data instance. If, according to MapSPAM, crop data was present in ecoregions with no CF values, a proxy CF from the nearest neighboring ecoregion of the same realm and biome was applied. For instance, the “Red Sea coastal desert” ecoregion, lacking specific CFs, was supplemented with data from the “Red Sea Nubo‐Sindian tropical desert.” Both ecoregions belong to the “Palearctic” realm and the “Deserts and xeric shrublands” biome, suggesting similar species densities and species–crop affinities and impacts. See the SI for full list of proxies applied.

The affected ecoregions represented marginal agricultural areas, comprising no more than 3% of the total cropland area by crop type in a country/region, thereby minimizing any potential assumption bias. The proxy CF method's validity was assessed by comparing it to an alternative approach where an average CF from all touching neighboring ecoregions was utilized. Both methods demonstrated negligible impact on the final aggregated country‐level CFs.

An absence of crop data in ecoregions with missing CFs prompts regions to be treated as zeros and excluded from the weighted average analysis. Where EXIOBASE regions are not covered in LC‐IMPACT, missing national CFs are replaced with a continental CF (e.g., European continent water stress CF as a proxy for Malta) or with approximates from similar countries (Chinese CFs for Taiwan). For the ROW regions of EXIOBASE, the relevant continental CFs from LC‐IMPACT were applied for calculating the baseline impacts. The harmonizing steps follow the recommendations of Mutel et al. (2019) for the handling of no data values.

### 2.7 Biodiversity impact accounts

Transformation of the environmental pressures quantified via the Leontief calculus in Section 2.3 is performed from both a consumption‐ and a production‐based perspective. $D_{cba}$, the consumption‐based footprint, and $D_{pba}$, the production‐based biodiversity impact account, are given in Equations (3) and (4), both characterized by the biodiversity LCIA matrix, **C**, through an element‐wise multiplication. Having created our disaggregated CFs, we have organized the characterization matrix, **C**, by crop and land use type. This organization allows for a more detailed and less sparse representation of biodiversity impacts, aligning with the logic of the EXIOBASE framework, where flows in S are distinguished by crop. Consequently, the matrix multiplication involves C having a column for each country/crop pair.

(3) $$D_{cba}=C⊙\mathrm{SLy}$$

(4) $$D_{pba}=C⊙F$$

## 3 RESULTS

### 3.1 The effect of spatial disaggregation on LC‐IMPACT characterization factors

**FIGURE 1:**
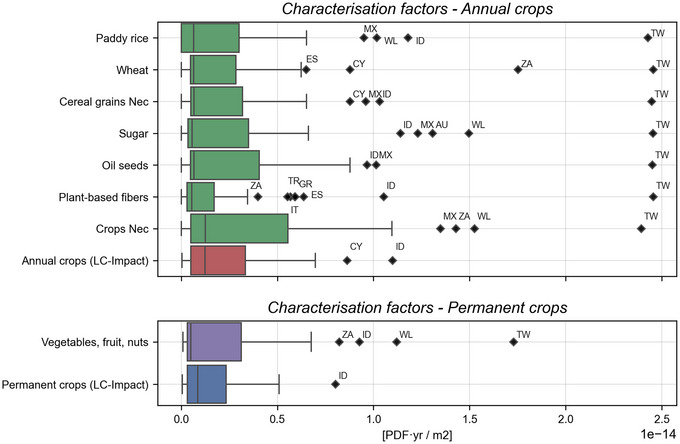
Differences in national land use characterization factor distributions post disaggregation for the Annual and Permanent crop categories in LC‐IMPACT and for the 49 EXIOBASE regions. Please see SI S1 for region codes and SI S2 for underlying data.

The distribution of the newly formed categories of national cropland CFs for the 49 Exiobase regions is presented in Figure 1. The eight national crop CFs (green and purple barplots) have been created via weighted sums of cropland land shares. The results reveal that national CFs aggregated in this way have distributions that diverge significantly between crop categories but also from CFs calculated as a weighted sum based on ecoregion land shares (red and blue barplots). The crop categories “Oil seeds,” “Crops Nec,” and “Vegetables, fruit, nuts” have wider distributions. It suggests the ecological damage of land use for their production has been underrepresented in a number of countries to date.

Crop categories with similar CF distributions to the LC‐IMPACT factors mask significant internal change at the country level. The production of sugar in Australia for instance is concentrated in ecoregions of greater sensitivity to land occupation than if predicted by ecoregion land shares. The Australian CF for sugar cropland is 1.31 × 10^−14^ PDF/m^2^, the third highest for sugar globally. The Annual crop CF for Australia in LC‐IMPACT is only the 18th largest however. The narrower distribution for plant‐based fiber CFs points to an overestimation of the crops' impacts.

Taiwan is a significant outlier in all crop categories. A level of uncertainty surrounds the Taiwanese CF results as national CFs are not provided in LC‐IMPACT. This could owe to a large level of uncertainty in the ecoregion CFs in the region or to a lack of taxonomic coverage. We construct CFs for Taiwan in any case and compare with the national CFs of China as a proxy.

The northern transitory climate regions of Canada, Russia, and Sweden all experience large increases in CFs across all crop categories. The countries accommodate ecoregions with large land surface areas in their northern territories. The northern ecoregions have lower land CFs due to lower levels of species richness and endemism (CAFF, 2013; IUCN, 2021). Both the climate and terrain limit the potential of the northern regions to support significant biodiversity rates. The same factors also hinder cropland expansion toward the north, resulting in the concentration of cropland in southern regions of these countries. Here, conditions are more compatible to crop production but also for supporting greater species diversity (Verones, 2021). Thus, a unit of land use in the south has a greater potential for biodiversity impacts while also being more likely to be used for agricultural purposes.

**FIGURE 2:**
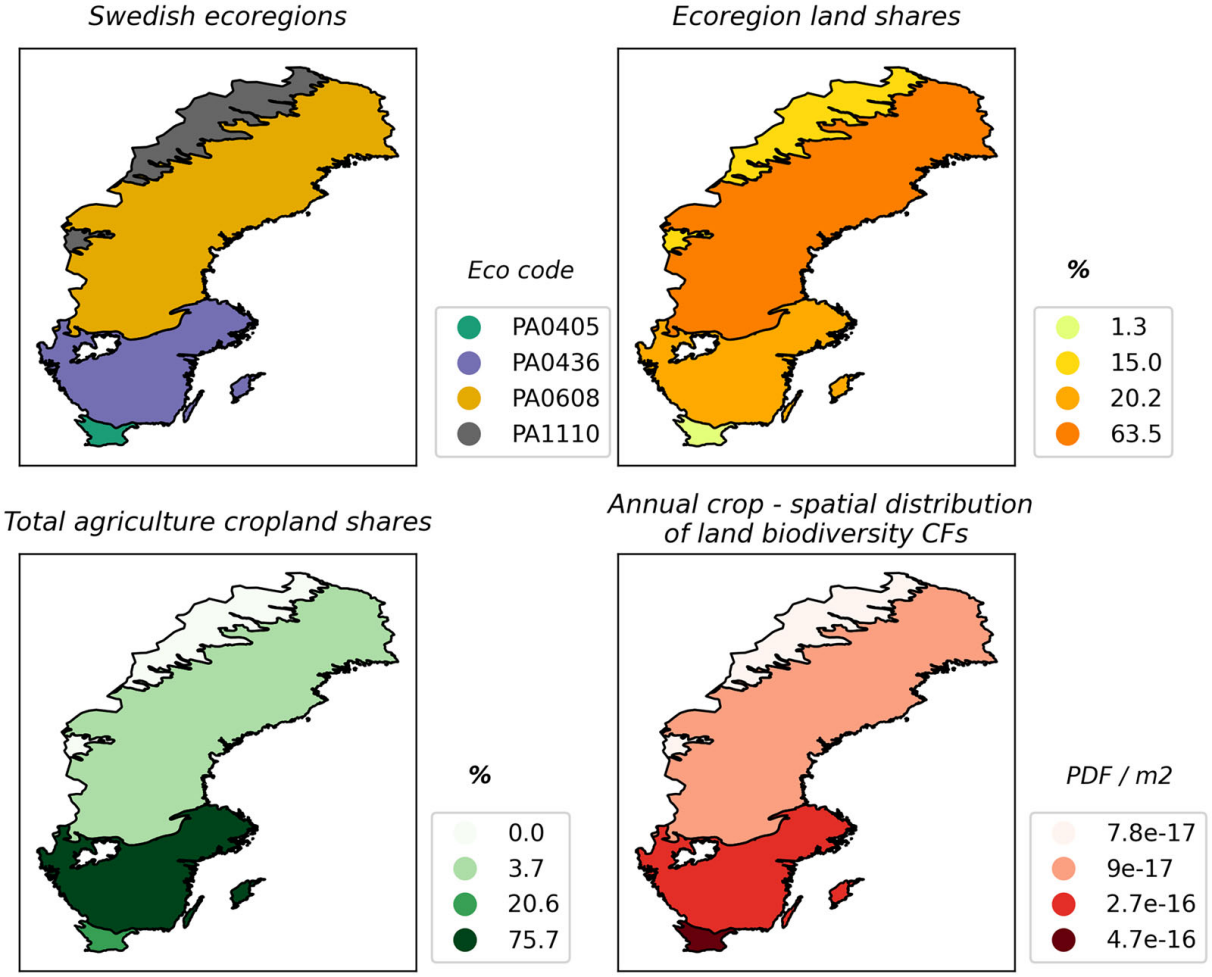
Four panel map of Swedish ecoregions analyzing the share of total land surface area in each ecoregion and the spatial distributions of annual cropland characterization factors and annual cropland area within the four ecoregion borders of Sweden. PA0608, Scandinavian and Russian taiga; PA0405, Baltic mixed forests; PA0436, Sarmatic mixed forests; PA1110, Scandinavian Montane Birch forest and grassland. Underlying data can be found in SI S2.

Figure 2 visualizes the contrasting distributions of ecoregion and cropland land shares in Sweden and the relative sensitivity of species in each ecoregion to annual cropland occupation. A concentration is found in the south, where 96.3% of total annual cropland exists in ecoregions comprising of just 21.5% of the total Swedish land surface area. The two ecoregions are also ecologically more sensitive to annual cropland occupation.

The ROW regions of the Americas, Asia, and Africa, already shown to be the origins of the largest shares of impacts related to agricultural land use (Chaudhary & Kastner, 2016; Verones et al., 2017), generally see increases in their respective spatially dissolved CFs generally. Decreases in CFs for central European countries can broadly be observed and there is less divergence in the EU‐27 compared to the variance in other regions. Full results are available in the SI.

### 3.2 Biodiversity accounts derived through default and modified characterization factors

The biodiversity impacts for land use and blue water consumption (in the SI) are presented categorically and independent of each other. We acknowledge the possibility for harmonizing the biodiversity effects from land and water use within LCA (Verones et al., 2015). Our research, however, seeks to outline differences in footprint results between spatially explicit and non‐explicit CFs and does not attempt to delve into LCA trade‐off style analysis of yield efficiencies and rainfed or irrigated production technologies. Normative assumptions for aggregating impacts from different stressors and taxa are avoided as a result.

#### 3.2.1 Production‐based accounts

**FIGURE 3:**
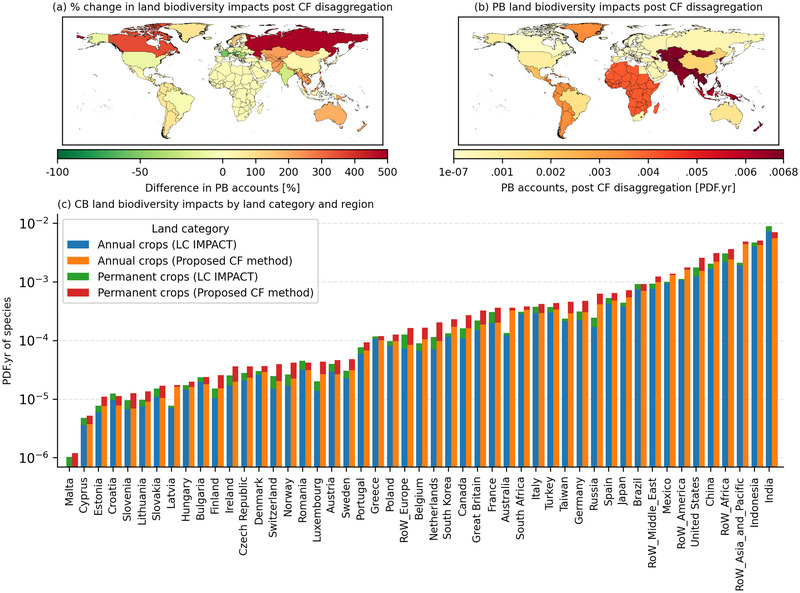
(a) The percentage change in production‐based (PB) biodiversity impacts for cropland occupation between national characterization factors aggregated via the proposed characterization factor method and LC‐IMPACT. (b) National PB biodiversity impacts for agricultural cropland occupation via the proposed disaggregated characterization factor method. (c) Comparison of national consumption‐based (CB) biodiversity impacts for agricultural cropland occupation via the proposed disaggregated characterization factor method and LC‐IMPACT. Underlying data can be found in SI S2.

The global land ecosystem consequences for servicing global final demand for agriculture products in 2020 was a PDF.yr of 0.0386. This means that 3.86% of the global species richness (of the taxa considered) may have been potentially lost in the given year due to crop production‐related environmental mechanisms. Applying the spatially explicit CFs increased the estimated impacts by 23.5%. Characterizing with the existing LC‐IMPACT CFs, India and Indonesia are the largest contributors to agricultural impacts but after our procedural reconstruction of the CFs, they are replaced by the ROW Asia (Figure 3). ROW Asia climbs from fourth to first place overall, increasing its production‐related accounts by 224% to 0.0068 PDF.yr.

Ecoregion land shares were a valid predictor of land impact intensities within the EU‐27 with some exceptions (France, Austria, and Sweden). Brazil and the United States saw only minor changes to their footprints. The sizable increase in CFs for the northern temperate regions predictably translate to large increases in national impacts. Swedish land impacts due to cereals production trebled, while Russian impacts for paddy rice production were underestimated by a factor of 10. In Australia, MapSPAM observes sugar and oil seed production in ecoregions of greater sensitivity than predicted via ecoregion land shares leading to increased impacts.

**FIGURE 4:**
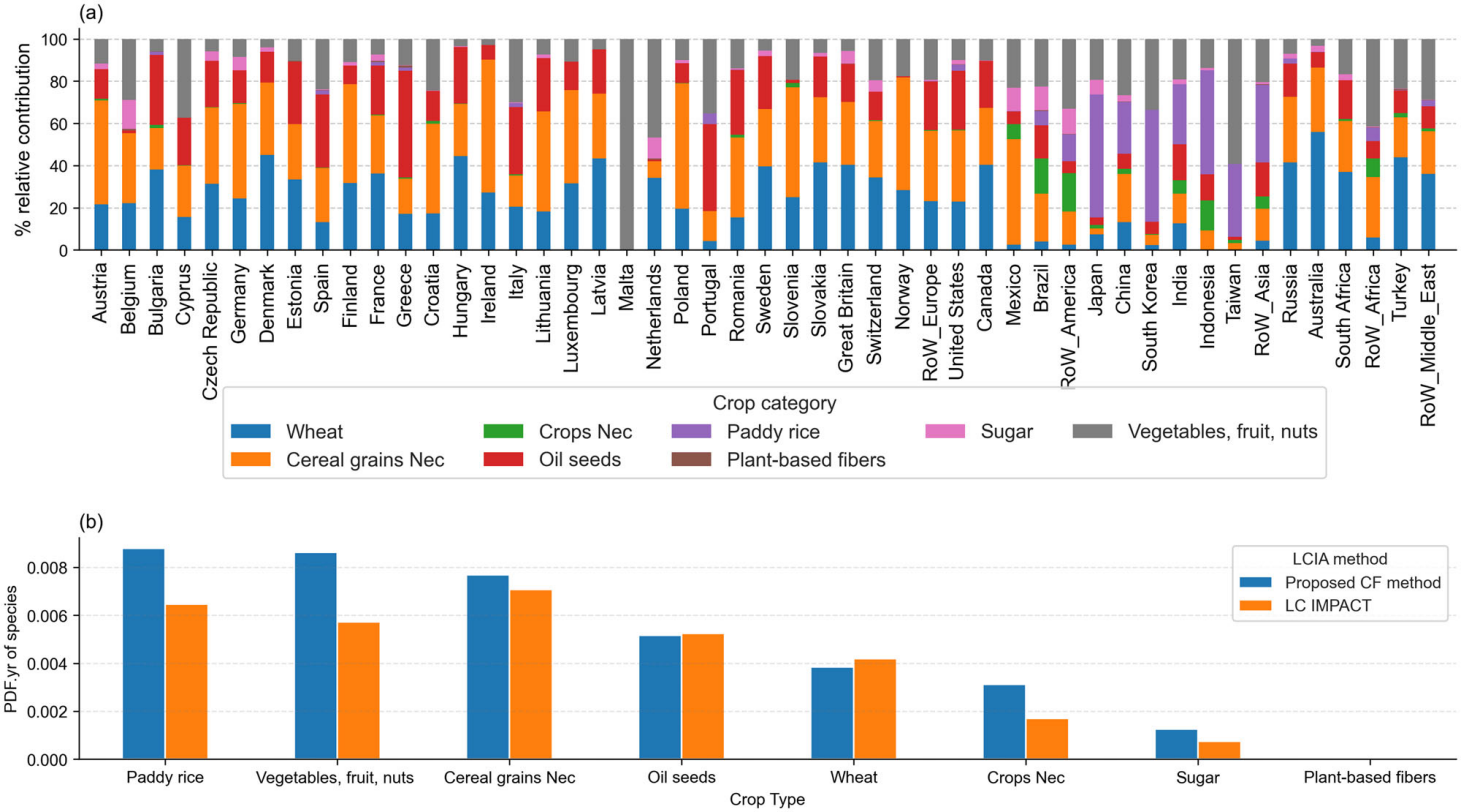
(a) Relative contributions of the eight EXIOBASE crop categories to the total production‐based biodiversity impacts of agricultural crop systems in the year 2020, by region. (b) Comparison of global absolute production‐based biodiversity impacts via LC‐IMPACT and the proposed characterization factor method. Underlying data can be found in SI S2.

Globally, aggregated ecoregion share CFs underestimate land‐related impacts from vegetables, fruit and nuts, paddy rice, cereal grains nec, crops nec, and sugar. CFs for both aggregation methods converge to describe global production‐based impacts for wheat and oil seeds but the global view masks large internal differences at the country level. Generally, crop categories have regional trends in impacts. Cereals and oil seed production represents over 80% of cropland ecosystem consequences in Europe. Paddy rice is the dominant cropland stressor in Asia (Figure 4.).

#### 3.2.2 Biodiversity footprints

India, Indonesia, and the ROW regions of Asia and Africa have the highest absolute biodiversity losses from a consumption perspective when aggregating CFs based on cropland shares. The majority of countries see a rise in their biodiversity footprints after applying the CFs aggregated in this manner. While both aggregation methods described European production‐based impacts similarly, European biodiversity footprints swell significantly when CFs are based on cropland share. Belgium's footprint, as a case in point, rose to 1.6 × 10^−4^ PDF.yr. An increase of 83 %. As expected, wealthy Western countries appear higher up the rankings tables on a consumption‐based biodiversity impacts metric. They are net importers of biodiversity impacts from global agriculture trade and thus increases were in line with expectations once agricultural exporting regions with dense species rich areas experienced large increases in impacts from a production perspective post disaggregation.

## 4 DISCUSSION

### 4.1 The uncertainty of aggregation approaches on biodiversity impacts

Previous work suggested that the largest uncertainty in the quantification of national biodiversity impacts lay with the use of national biodiversity CFs (Koslowski et al., 2020). The results align with this hypothesis, in that we have seen significant divergence in both the CFs in LC‐IMPACT and on the national biodiversity impacts due to applying a different aggregation approach. The proposed approach increased global production‐based biodiversity impacts by 23.5%.

Other footprint studies have consistently pointed to the low level of regional data for countries not explicitly covered in the MRIO databases as a significant limitation (Cabernard & Pfister, 2021; Moran et al., 2016). ROW regions are responsible for the largest share of land‐related biodiversity impacts owing to their higher rates of species richness and endemism as we and others have seen (Bjelle et al., 2021; Lenzen et al., 2012; Verones et al., 2017).

The detailed country‐level granularity of Eora26 (Lenzen et al., 2013) is likely to provide a more accurate picture of aggregated agricultural impacts globally and for individual countries within the ROW regions when used in conjunction with the proposed LCIA aggregation method. However, Eora26 offers limited insight into specific crop impacts due to the aggregation of agricultural production into a single sector and use of broader land use stressor categories. This aggregation obscures notable trends in individual countries that can be revealed through more detailed sector and crop resolution (e.g., the significant impacts of sugar in Australia uncovered). Thus there is a trade‐off again between country and sector resolution, a common problem in EE‐MRIO analysis.

Bjelle et al. (2021) applied a spatially disaggregated form of EXIOBASE, version 3rx (Bjelle et al., 2019), for investigating this spatial problem. They concluded that regions in Asia and Africa should be represented in a finer level of spatial detail to avoid aggregation errors. Cabernard and Pfister (2021)'s approach was to merge the Eora and EXIOBASE databases, increasing the country spatial resolution from 49 regions to the resolution of Eora (189) while maintaining the high sector detail of EXIOBASE.

They found EU consumption‐based footprints for land use to increase by between 2% and 6% on footprints calculated using EXIOBASE 3 for the years 1995 to 2015. Our study observed an increase of 23.2% for EU consumption‐based biodiversity footprints for the year 2020. It suggests biodiversity CFs and their aggregation method deserve as much attention for determining biodiversity impact outcomes as increasing the country‐level granularity in EE‐MRIO models.

Both issues are important; therefore, the choice of the “best” MRIO database for biodiversity impact studies should depend on the specific goals of the study. In this case, the greater agricultural sector resolution of EXIOBASE 3 enabled us to test the elementary flow aggregation technique at the highest available resolution and to identify crop‐specific biodiversity impact hotspots.

While the biodiversity impact findings are interesting in themselves, the underlying issues with aggregation are not exclusive to any single MRIO database and LCIA combination. Even for countries where EXIOBASE data was available, substantial changes in CFs and impacts underscore the perils and importance of normative aggregation choices. These issues also apply to LCA and LCI datasets, where the geographical resolution of agricultural inputs and outputs often does not extend beyond the national level. The implications of the results could also extend to the use of other LCIA methods in LCA where CFs are also spatially dependent such as human health damage factors from local air pollutants but it is unclear whether it would increase or reduce impacts.

These issues can be more easily rectified in LCA datasets if practitioners are aware of the uncertainty introduced by upward aggregation. An LCI dataset can be re‐engineered to introduce spatial resolution applicable to the system boundary of a study. If not at the ecoregion level, then at a subnational level, whereby a similar aggregation technique as applied here could be applied at an even greater resolution. Ideally, avoiding aggregation altogether would be preferable. Avoiding aggregation is more challenging for studies using EE‐MRIO due to the lack of economic data at the scales necessary to avoid such practices. The next step for EE‐MRIO models should be to move beyond the national level to a subnational scope. Work on this is already under way (Sun et al., 2018).

The results of these studies are limited by the high uncertainty in the input data, making it challenging to quantify findings. For example, the MRIO model by Cabernard and Pfister (2021) relies on proxy data for ROW countries from Eora26 and FAOSTAT. Similarly, our study's input data also has uncertainties. Model confidence in MapSPAM varies by crop and country based on data sources (International Food Policy Research Institute, 2024). Combining an LCIA model (LC‐IMPACT) with a more spatially explicit crop dataset, both with their independent set of modeling assumptions, raises the question of the scientific exactness of bridging the two models from an ecological perspective. CFs should be developed within LCIA models rather than merging two characteristically different models together. Further, it is questionable whether national CFs can effectively capture ecosystem consequences for impacts that are site specific and locally rooted.

### 4.2 Crop impacts

Similar to previous studies on the impact of land occupation on food systems (Chaudhary & Kastner, 2016), our results reveal that extensive crops, such as cereals, which dominate global agricultural land footprints (FAO, 2024), are also responsible for the largest share of terrestrial ecosystem damage (Figure 4). While crop extensiveness indicates land occupation dynamics, land transformations can account for up to 57% of total biodiversity impacts (Verones et al., 2015). Land transformations refer to the process of converting natural habitats, into other land uses. Food crops linked to tropical deforestation such as coffee and cocoa will have larger global impacts despite smaller pressure footprints because of the transformation of virgin ecosystems to establish their production (Kastner et al., 2021; Pendrill et al., 2019).

Internal country dynamics are more opaque. Large CF fluctuations within countries and between crops show the importance of crop regionalization and category disaggregation. Weighting regional CFs via ecoregion land shares, independent of crop or activity are imprecise proxies for true spatial production data and have possibly underestimated land impacts to date. The disparity between national CFs aggregated via ecoregion land shares or elementary flows demonstrates the need for spatial alignment between LCIA models and inventory flows.

Divergence in crop CFs amongst countries raises the potential of international trade for reducing crop‐related impacts if otherwise local demand was met by domestic production only. While numerous studies have concluded that international trade is responsible for between 20% and 30% of global biodiversity impacts (Bjelle et al., 2021; Lenzen et al., 2012; Wilting et al., 2017), Kastner et al. (2021) estimated that international trade has a net positive effect on biodiversity due to land sparing from the export of certain food crops from countries with higher yield capabilities and lesser biodiversity impact intensities. Such conclusions, when only considering one impact pathway (land use) need to be considered with caution. Comparing “cereals nec” production of the United States with that of the second largest exporting region in 2010, ROW America, the United States land impact intensity is seven times lower. However, wetland impacts in the United States from “cereals nec” production post CF disaggregation increase by 544% and its impact intensities per million euro of crop consumption are over 350 times greater than those of ROW America.

The nexus of food production and biodiversity necessitates trade‐offs where maximizing yield efficiencies requires the introduction of irrigation technology, increasing blue water consumption impacts but reducing land impacts through land sparing and vice versa. Potentially, a symbiotic relationship between the two is possible by optimizing the preferential growing locations of crops and merging with demand side policies as suggested in the scenario modeling by Leclère et al. (2020). Specialization and intensification bring additional ecosystem concerns like water scarcity and a string of social and geopolitical concerns around food security (Fischer et al., 2017).

### 4.3 Limitations

It is imperative that pasture cropland be included in future analysis with previous work apportioning significant impacts to cattle rearing for example (Marquardt et al., 2019). Land fragmentation and land use intensity practices are not accounted for here. Incorporating fragmentation dynamics predicts higher median per‐area impacts of land use compared with the standard species‐area c‐SAR model (Kuipers et al., 2021). The availability of yield data in MapSPAM and farming inputs data from FAO (2022) could allow for land management analysis but further development of biodiversity responses to such intensities beyond the novel work conducted by Chaudhary and Brooks (2018) is required.

## 5 CONCLUSION

Using a spatially resolved agricultural production model, we disaggregated the number of LC‐IMPACT cropland CFs available for land use and created national CFs weighted by cropland land areas rather than ecoregion land areas. The results revealed an increase in global production‐based biodiversity impacts of 23.5% and 17.5% for land use and blue water consumption, respectively. Our results are evidence that broad cropland categories of land use and current upward aggregation techniques for native scale CFs are a poor proxy for describing country‐level ecosystem damage from agricultural production. Consequentially, avoiding aggregation if possible in LCA studies should be prioritized. Otherwise, a harmonized approach to aggregation is required, if studies on biodiversity impacts are to be compared effectively. The large variations in CFs between countries demonstrate the pressing need for regionalization of MRIO models beyond the national scale if the heterogeneity of biodiversity impacts are to be fully realized. The significant diversity in CFs across different crops highlights the importance of conducting further research on impact assessments for various cropland categories. This will not only enhance the accuracy of crop‐to‐crop comparisons but also improve the overall understanding of LCIA methods.

When applying biodiversity characterization factors, it is recommended that LCA practitioners use activity data that is, at the very least, on the same spatial scale as the native CFs in the LCIA models. Avoiding the aggregation of native scale PDF scores would remove many of the uncertainties discussed in this study. However, if applying country‐level biodiversity CFs, LCA professionals must be aware of the implicit uncertainty this introduces, and this must be clearly communicated to the involved stakeholders and decision makers. Additionally, LCIA practitioners must seek to improve national‐level CFs if biodiversity footprint analyses with current MRIO tables are to be actionable.

## CONFLICT OF INTEREST STATEMENT

The authors declare no conflicts of interest.

## Supporting information

Supporting information is linked to this article on the JIE website:
**Supporting Information S1**: This supporting information details the EXIOBASE region codes (1.), outlines the Leontief calculus (2.), describes the assumptions underlying the mapping between MapSPAM and EXIOBASE products (3.), and presents method (4.) as well as results (5.) for tailored blue water consumption characterization factors.

**Supporting Information S2**: This supporting information provides: data used for creating the figures in the main text, the mapping between EXIOBASE and MapSPAM, the proxy treatment of LC‐IMPACT and MapSPAM data, the per‐country characterization factors for water and land pressures.

## Data Availability

The data that support the findings of this study are available in the supporting information of this article. Data underlying this generated data are available at https://zenodo.org/records/5589597 (EXIOBASE), https://doi.org/10.7910/DVN/SWPENT (MapSPAM), and https://zenodo.org/record/6200606 (LC‐IMPACT); auxiliary data is cited properly where due. The Python code used to generate the findings of this study is available from the first author upon reasonable request.
