# Supplementary material for: Examining global biodiversity accounts: Implications of aggregating characterization factors from elementary flows in multi-regional input–output analysis
Source: J Ind Ecol. 2024 Oct 8;28(6):1422–34. doi: 10.1111/jiec.13556 (PMC11667647; doi:10.1111/jiec.13556)
Supplement: Supplementary file 1 — Supporting information is linked to this article on the JIE website: Supporting Information S1: This supporting information details the EXIOBASE region codes (1.), outlines the Leontief calculus (2.), describes the assumptions underlying the mapping between MapSPAM and EXIOBASE products (3.), and presents method (4.) as well as results (5.) for tailored blue water consumption characterization factors. [file 44498_2024_2806008_MOESM1_ESM.docx]

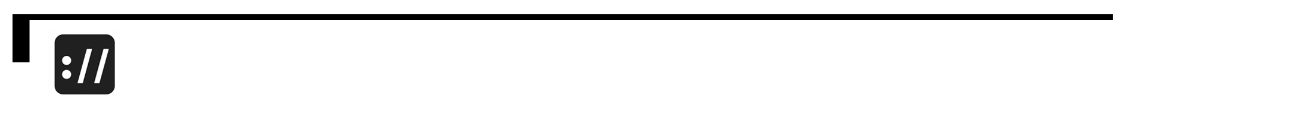


SUPPORTING INFORMATION FOR:

Davin, K., Koslowski, M., Dorber, M., Hertwich, E. (2024). Examining global biodiversity accounts: Implications of aggregating characterization factors from elementary flows in Multi-Regional Input-Output Analysis. *Journal of Industrial Ecology.*


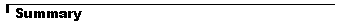


This supporting information details the EXIOBASE region codes (1.), outlines the Leontief calculus (2.), describes the assumptions underlying the mapping between MapSPAM and EXIOBASE products (3.), presents method (4.) and results (5.) for tailored blue water consumption characterization factors.


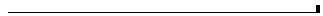


## EXIOBASE region codes

| **Code** | **Full region name** |  | **Code** | **Full region name** |
| --- | --- | --- | --- | --- |
| AT | Austria |  | KR | South Korea |
| AU | Australia |  | LT | Lithuania |
| BE | Belgium |  | LU | Luxembourg |
| BG | Bulgaria |  | LV | Latvia |
| BR | Brazil |  | MT | Malta |
| CA | Canada |  | MX | Mexico |
| CH | Switzerland |  | NL | Netherlands |
| CN | China |  | NO | Norway |
| CY | Cyprus |  | PL | Poland |
| CZ | Czech Republic |  | PT | Portugal |
| DE | Germany |  | RO | Romania |
| DK | Denmark |  | RU | Russia |
| EE | Estonia |  | SE | Sweden |
| ES | Spain |  | SI | Slovenia |
| FI | Finland |  | SK | Slovakia |
| FR | France |  | TR | Turkey |
| GB | Great Britain |  | TW | Taiwan |
| GR | Greece |  | US | United States |
| HR | Croatia |  | ZA | South Africa |
| HU | Hungary |  | WA | Rest of World Asia and Pacific |
| ID | Indonesia |  | WE | Rest of World Europe |
| IE | Ireland |  | WF | Rest of World Africa |
| IN | India |  | WL | Rest of World Americas |
| IT | Italy |  | WM | Rest of World Middle East |
| JP | Japan |  |  |  |

## Basic Leontief calculus

The standardised methodology for IO analysis is that of the Leontief inverse calculus (Leontief, 1936, 1970). Letting upper case letters represent matrices and lower-case letters represent vectors, IO tables are composed of an *mn × mn* transaction matrix **Z**, an *mn × k* final demand matrix **Y**, and the *f × mn* environmental satellite account of the economy, **F**, where *m* is the number of regions, *n* the number of product sectors, *k* the number of final demand categories, and *f* the number of stressors. The *mn × 1* total industrial output requirements for a country/region are then given by equation (1) where **i** is a summation vector of ones.

1. $\boldsymbol{x = Zi +Yi}$

All non-agriculture and food-related product demand is set to zero in ***Y***. The technology coefficient matrix, **A**, is derived from $\boldsymbol{A = Z}{\hat{\boldsymbol{x}}}^{\boldsymbol{-1}}$*.* Rearranging the terms yields:

1. $\boldsymbol{x=}\left( \boldsymbol{I-A} \right)^{\boldsymbol{-1}}\boldsymbol{y}$

where **I** is an identity matrix, and $\boldsymbol{L}\boldsymbol{=}\left( \boldsymbol{I-A} \right)^{\boldsymbol{-1}}$ is known as the Leontief inverse, with dimensions *mn×mn*. This equation indicates the total industrial output of the economy for an arbitrary vector of final demand, ***y***, under the assumption of a constant linear economy (Miller et al., 2009) (Leontief, 1936, 1970)

Given the total environmental pressure footprint, ***F***, the stressor coefficient matrix, $\boldsymbol{S =F}{\hat{\boldsymbol{x}}}^{\boldsymbol{-1}}$, is the normalized environmental satellite, showing the environmental inputs per unit of monetary output of a sector. The diagonal blocks of ***S****,* ***L****,* ***Y*** represent the domestic economy, while the off-diagonal blocks represent the traded parts of the economy in a multi-regional input-output (MRIO) system. The off-diagonal parts of the ***S*** matrix are zero as there are no traded impact multipliers of production.

Production-based environmental accounts are equal to the factors of the production matrix $\boldsymbol{F}$. Typically, the consumption-based environmental footprint combines the environmental footprint in terms of the final demand vector, $\boldsymbol{y}$, with the direct pressure matrix, $\boldsymbol{F}_{\boldsymbol{y}}$, resulting from the final consumption across all final demand categories. For simplicity, we analyse here impacts purely related to the production of agriculture to service the final demand in the global economy and exclude the direct impacts from final demand:

1. $\boldsymbol{E=SLY}$

## MapSPAM mapping to EXIOBASE crop categories

Nuts were included in the EXIOBASE Crops Nec category rather than “Vegetable, Fruit, Nuts” due to the category being aggregated within the “Rest of crops” category in MapSPAM. Nut production in terms of land area comprises of a small proportion of overall cropland use in both “Vegetable, Fruit, Nuts” or “Crops Nec” (FAO, 2024) and thus this mapping issue is not expected to have a material impact on the aggregation of ecoregion EFs to the national level for “Vegetable, Fruit, Nuts” or “Crops Nec”.

The choice was made to group Cotton production data from mapSPAM within the Plant based Fiber category of EXIOBASE. Cotton could just as easily have been placed in the Oilseed category due to the production of two different products from the same crop, lint (a fiber) and cottonseed (an oilseed). For simplicity here, we mapped at a one-to-one relationship between MapSPAM and EXIOBASE crop category. But another approach could have been to allocate land use between both plant-based fibre and oilseed based on mass allocation or economic allocation.

The "Vegetables, fruit, nuts" category is classified as permanent crops in EXIOBASE. Following this classification, we created aggregate CFs for "Vegetables, fruit, nuts" using the native Permanent Crop CFs from LC-IMPACT and the relevant disaggregated crop categories from MapSPAM. However, according to the Indicative Crop Classification (Version 1.1) (UN, 2021), while most fruits and nuts are permanent crops, vegetables are considered temporary (annual) crops. This discrepancy impacts the characterization of biodiversity impacts, as the application of Permanent Crop CFs to vegetables could result in underestimation or overestimation of their actual impacts. This is further influenced by the location-specific elementary flows in MapSPAM, which may skew the results due to the inclusion of the fruit category.

Generally, the per-unit impacts of annual cropland are higher than those of permanent cropland, according to national CFs in LC-IMPACT. This suggests that the impacts from vegetables have been underestimated in this study. However, disaggregating EXIOBASE to reflect these distinctions was beyond the scope of this research. Therefore, further research is needed to accurately quantify this estimation.

## Blue water consumption characterisation factor methodology

For Blue water consumption, the older MapSPAM data for the year 2010 was used in this modelling as it was the latest dataset available at the time of analysis. The data was merged with EXIOBASE v3.8.1 and the pxp tables for 2010. Other than this distinction all other EXIOBASE modelling parameters remained the same as what was discussed in the methods section of the main body.

For water stress, CFs are constructed at the watershed `catchment' level and impacts are modelled based on the change in wetland area due to water consumption. Native CFs for blue water consumption are aggregated by weighting total blue water consumption occurring per watershed independent of crop or activity. Water CF construction requires the addition of a spatially explicit water dataset to translate crop production volumes in tonnes to blue water irrigation demands in m3.

The water data provided by Pfister and Bayer (2019) has global coverage for 160 crops, with blue water consumption per tonne of crop produced and total blue water consumption datasets available for each crop in over 12'000 watershed units for the year 2000. The 42 aggregated crops of mapSPAM (International Food Policy Research Institute, 2019) and 160 crops of Pfister and Bayer (2019) were merged into the following nine distinct agricultural categories according to the Food and Agriculture Organisation (FAO) and EXIOBASE (Stadler et al., 2018) crop taxonomy: “Paddy Rice”, “Wheat”, “Cereal grains not elsewhere classified (nec)”, “Sugar”, “Oil seeds”, “Plant-based fibers”, “Vegetables, fruit and nuts”, “Crops not elsewhere classified (nec)”' and “Fodder crop”. mapSPAM does not contain production statistics for fodder crops but the Pfister water dataset has total blue water consumption data for the crop in the year 2000. Hence, the blue water consumption volume for 2000 was used as a proxy.

The blue water elementary flows were calculated by combining the blue water consumption per tonne of produced crop data for the year 2000 of Pfister and Bayer (2018) with the irrigated crop production quantities of mapSPAM for the year 2010 (International Food Policy Research Institute, 2019). The resulting data is to act as a proxy for elementary blue water crop consumption in the year 2010 in the absence of primary data sets for the elementary flow. For data gaps in polygons where mapSPAM assesses irrigated crop production to take place but no water consumption data exists, national average blue water consumption per tonne of crop produced data for the crop in question was applied. In contrast, if blue water data exists for a polygon but crop data in mapSPAM suggests no crop production to exist, it is concluded that no consumption occurs in the polygon for this crop. The calculation of national water CFs for the nine disaggregated annual and permanent crop types are presented in equation S1, with variables listed in Table S1. For watersheds not covered in LC-IMPACT but present in the Pfister and Bayer (2018) dataset, existing national CFs were applied for missing watersheds within a country's boundaries. For the native scale CFs, only surface water consumption effects were considered due to the low level of robustness for groundwater-fed wetlands data in LC-IMPACT (Verones et al., 2020).

The application of the irrigated crop dataset from MapSPAM allowed for the distinction between blue and green water consumption for agricultural production. If water requirements are fully met by green water consumption, blue water biodiversity impacts are assumed to be zero and this methodological reasoning was applied across all crop categories and countries. For example, “wheat” and “paddy rice” are extensively grown in rainfed regions around the world (Molden et al., 2011) (Seck et al, 2012) and their production in a handful of countries produces no blue water related biodiversity impacts.

Equation S1 – Blue water consumption characterization factor formation

$${WC}_{jx}= \sum_{h=1}^{n} \frac{Q_{hjx}\times K_{hjx}}{\sum_{h=1}^{n} Q_{hjx}\times K_{hjx}}\times C_{hx}$$

| Variable name | Description | Unit |
| --- | --- | --- |
| C_hx_ | LC-IMPACT blue water consumption CF for polygon h | PDF / m^3^ |
| WC_jx_ | Blue water consumption CF for crop j in country/ROW region x | PDF / m^3^ |
| Q_hjx_ | Irrigated production volume of crop category j in polygon h | Million metric Tonnes (MT) |
| K_hjx_ | Blue water consumption per tonne of crop j in polygon h | m^3^ / MT |
| n | Number of polygons in country/ROW region x |  |

Table S1 - Variables required in equation 1 for the aggregation of native scale water stress characterisation factors to the national level.

##
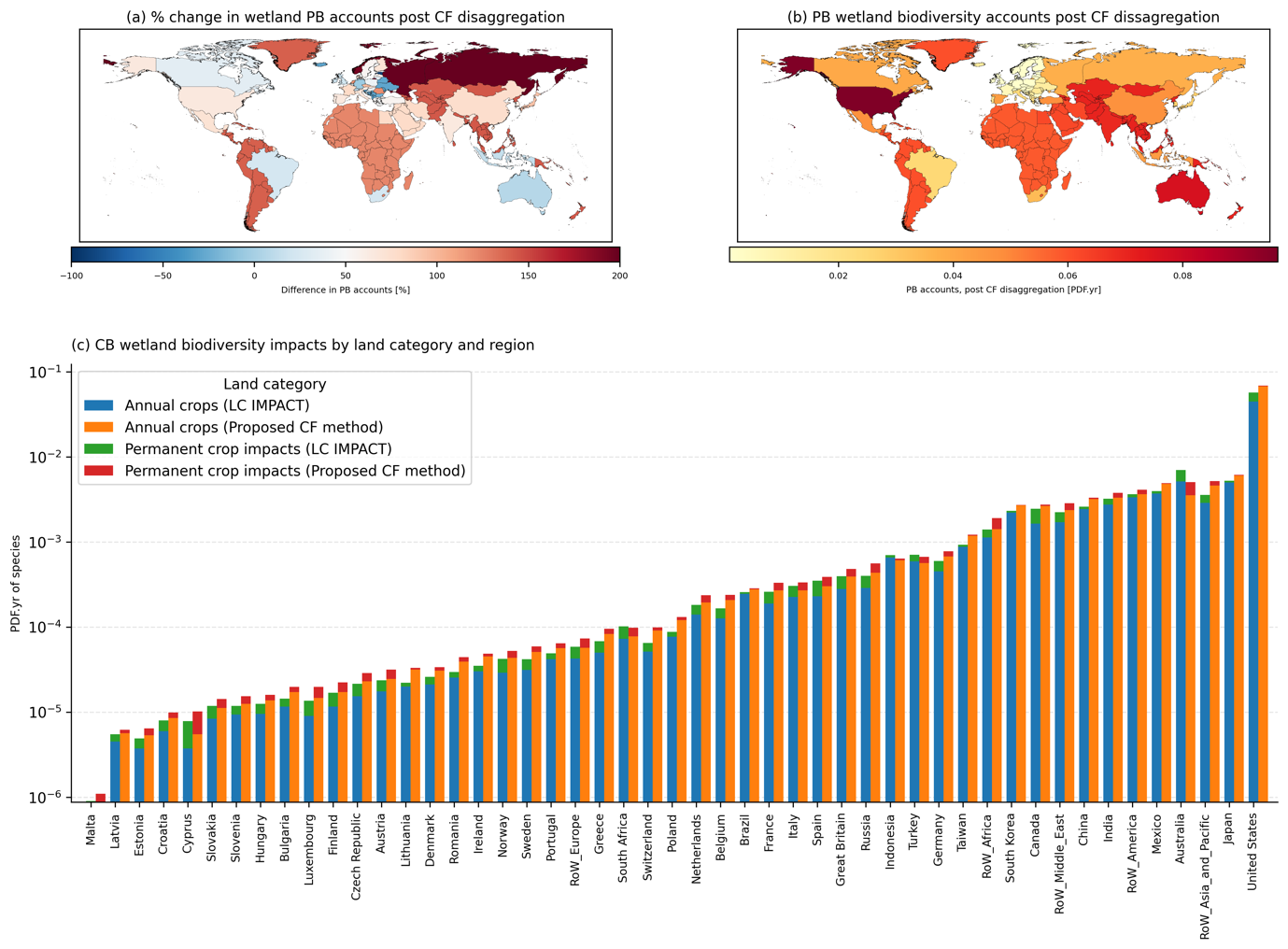
Blue water consumption – Results

Figure S1: (a) the % change in production-based (PB) wetland biodiversity impacts for blue water crop consumption between national CFs aggregated via the proposed characterisation factor method and LC-IMPACT. (b) National production based (PB) wetland biodiversity impacts for blue water crop consumption via the proposed disaggregated characterisation factor method. (c) Comparison of national consumption based (CB) wetland biodiversity impacts for blue water crop consumption via the proposed disaggregated characterisation factor method and LC IMPACT.

Significant variation in country/continental CFs are observed for the eight tailored crop categories. A single aggregated country/continent level CF based on shares of total blue water consumption fails to effectively describe crop specific dynamics transpiring within a country's borders. Water impacts related to wheat and rice production have likely been overestimated to date due to the absence of explicit datasets on rain-fed and irrigated production. The US, a major producer of both crops, observes a reduction in impact intensities of 31% and 60% for wheat and rice production respectively based on where and to what degree irrigated crop production occurs. Changes to CF impact intensities in the US and Australia have global implications for biodiversity as their wetland habitats are affected by blue water withdrawals at greater rates that compare in orders of magnitude. The blue water CF in LC-Impact for the US is 1.15 E-12 PDF/m2 in contrast to the average regional CF of 9 E-14 PDF/m2. Post disaggregation, the two countries remain as ecological damage hotspots. In the US case, “oil seed” crops, “sugar” crops and “crops nec” see increased biodiversity impacts, with impacts from “crops nec” deviating substantially to 7.41 E-12 PDF/m2.

Spatially explicit blue water data is especially important in the Russian case with “wheat”, “paddy rice” and “vegetable, fruit and nuts'” production impact intensities increasing by 445%, 884% and 210% respectively. However, “oil seeds”, “plant-based fibers”, and “crops nec” are grown in ecologically less sensitive regions in Russia and have declining impact intensities. Figure S2 visualises the spatial distribution of CFs and blue water consumption for two crop categories: “cereal grains nec” and “paddy rice”, within Russia. It details the large deviation in locations and blue water consumption intensities for both crops, exposing the issue with single aggregated impact factors for characterising pressure footprints.


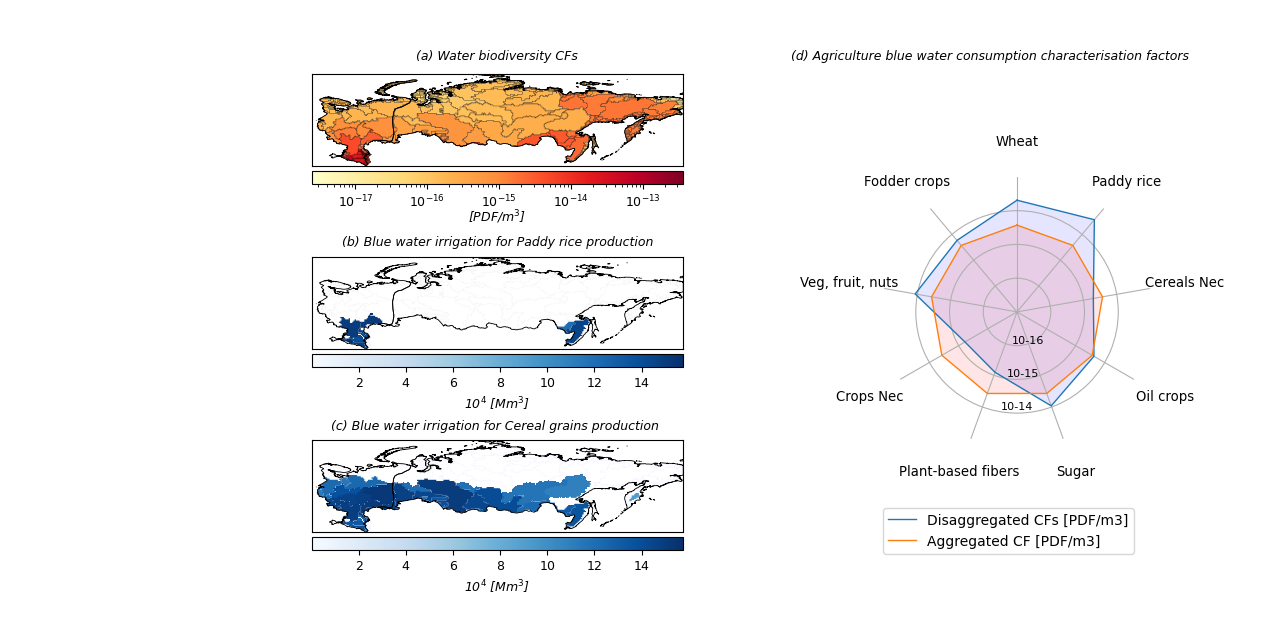


Figure S2: (a) Distribution of LC-IMPACT blue water characterization factors in Russia, (b) the distribution of blue water demand for paddy rice production in Russia in 2011, (c) the distribution of blue water demand for wheat production in Russia in 201, and (d) The change in biodiversity intensities for blue water consumption characterisation factors in Russia when disaggregating the annual crop category in LC-IMPACT and aggregating by elementary flows of blue water consumption.

The large continental ROW regions suffer similarly from the lack of spatial and categorical detail in LC-Impact. In tropical ROW regions, crops recognized for drawing large irrigation footprints are significantly underrepresented in terms of biodiversity impact intensities. This goes for rice production in ROW Asia, vegetable and fruit production in ROW Americas and fodder crops in ROW Africa).

Partitioning the single CF into eight crop categories and aggregating via crop specific locations and elementary flows increases the global wetland biodiversity impacts of cropland by 17.5%. Global burdens are driven by water consumption in the US, responsible for 81% of global impacts. The result is expected as its wetland CFs are orders of magnitude higher than most countries/regions included. The four regions of the US, Australia, ROW Asia and India are accountable for over 90% of impacts globally. The US's footprint increased from a PDF score of 0.08 to 0.096 for the year 2010. The increase was partly due to the acute impacts for the “crops nec” category after mapSPAM modelled growing regions to be concentrated in the highly sensitive wetland areas of the south-eastern seaboard. The US, like many countries, experience large fluctuations between crop categories. While the impacts from “crops nec” were acute, the distribution and spatial demand for blue water irrigation in the US for “wheat” and “vegetables, fruit and nuts” concluded that previous estimates of respective crop impacts were overstated. While diverging outcomes in the US resulted in broadly increasing wetland impacts overall, the opposite is true in Australia, where fluctuating crop impacts result in an overall decline in impacts.

Large spatial differences in crop locations and irrigation demands produce the largest divergence in Russia. Figure S2 illustrates the spatial distribution of blue water irrigation for “paddy rice” and “cereals nec” in Russia and the contrasting impacts on resulting Russian CFs. The outcome of greater spatial resolution in Russia's case is substantially increased wetland biodiversity consequences. “Vegetable, fruit and nuts” production is an extensive driver of impacts internationally, dominating biodiversity footprints in many countries. If irrigated rice production exists in a region, then it is likely the crop is the prevalent wetland stressor as is the case with South Korea, Japan, Russia and ROW Asia. Discounting the US impacts, wetland impacts from global wheat, rice, sugar and “Vegetables, fruit and nuts” production have been underrepresented, while the impacts from “fodder crops”, “oil seeds” and “crops nec” have likely been overstated. ROW regions containing extensive land areas, traversing several climates, growing conditions and levels of aridity all displayed noticeable variation in their respective ecosystem consequences post disaggregation. A comparison of blue water-associated production-based wetland biodiversity impacts per aggregation method and crop type is shown in Figure S3.

The temporal mismatch between the primary water consumption data from Pfister and the production year modelled in the study (2010) is acknowledged. Combining two different data sets spanning two time periods to calculate elementary crop blue water consumption in 2010 adds a level of uncertainty to the results that is not quantified. Assuming primacy of the Spatial Production Allocation Model data over the Pfister and Bayer blue water dataset when mismatches occurred in crop locations was done mainly because it was constructed for the year of our analysis and is a newer spatial model and not necessarily because of the superior underlying scientific rigour of the model itself. \par


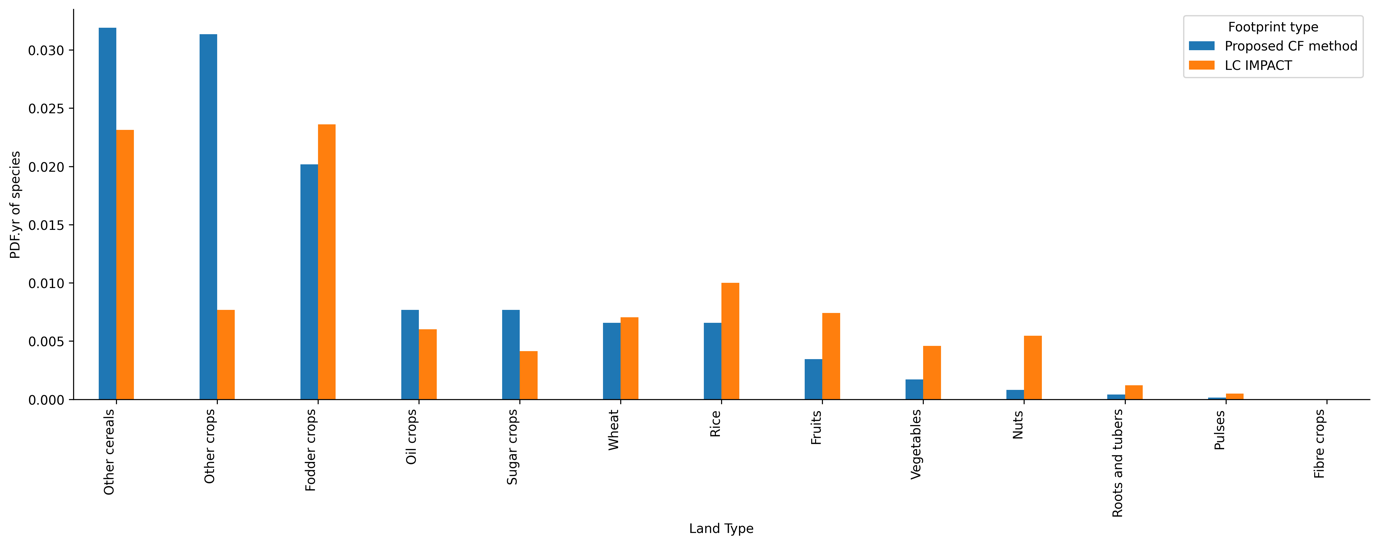


Figure S3: Comparison of global absolute Production Based (PB) wetland biodiversity impacts for blue water crop consumption via LC IMPACT and the proposed CF method.

## References

International Food Policy Research Institute, 2019, "Global Spatially-Disaggregated Crop Production Statistics Data for 2010 Version 2.0", <https://doi.org/10.7910/DVN/PRFF8V>, Harvard Dataverse, V4

Molden, D., Vithanage, M., Fraiture, C. de, Faures, J.M., Gordon, L., Molle, F. and Peden, D. 2011. Water availability and its use in agriculture. Treatise on Water Science 4: 707-732. <https://doi.org/10.1016/B978-0-444-53199-5.00108-1>

Papa Abdoulaye Seck, Aliou Diagne and Samarendu Mohanty et al. Crops that feed the world 7: Rice. *Food Sec.*2012. Vol. 4(1):7-24. DOI: [10.1007/s12571-012-0168-1](file:///C:\Users\maximiko\AppData\Local\Box\Box%20Edit\Documents\Bcz7kLGojEWIDH4iq4ToYQ==\10.1007\s12571-012-0168-1)

Pfister, Stephan; Bayer, Peter (2019), “Water consumption of crop on watershed level (blue and green water, uncertainty, incl. shapefile) and monthly irrigation water consumption”, Mendeley Data, V3, DOI: [10.17632/brn4xm47jk.3](file:///C:\Users\maximiko\AppData\Local\Box\Box%20Edit\Documents\Bcz7kLGojEWIDH4iq4ToYQ==\10.17632\brn4xm47jk.3)

Stadler, K., Wood, R., Bulavskaya, T., Södersten, C.-J., Simas, M., Schmidt, S., Usubiaga, A., Acosta-Fernández, J., Kuenen, J., Bruckner, M., Giljum, S., Lutter, S., Merciai, S., Schmidt, J.H., Theurl, M.C., Plutzar, C., Kastner, T., Eisenmenger, N., Erb, K.-H., de Koning, A. and Tukker, A. (2018), EXIOBASE 3: Developing a Time Series of Detailed Environmentally Extended Multi-Regional Input-Output Tables. Journal of Industrial Ecology, 22: 502-515. DOI: <https://doi.org/10.1111/jiec.12715>

Verones, F, Hellweg, S, Antón, A, et al. LC-IMPACT: A regionalized life cycle damage assessment method. J Ind Ecol. 2020; 24: 1201–1219. DOI:  <https://doi.org/10.1111/jiec.13018>

Yu, Q., You, L., Wood-Sichra, U., Ru, Y., Joglekar, A. K. B., Fritz, S., Xiong, W., Lu, M., Wu, W., and Yang, P.: A cultivated planet in 2010: 2. the global gridded agricultural production maps, Earth Syst. Sci. Data Discuss., https://doi.org/10.5194/essd-2020-11, in review, 2020. DOI: [10.5194/essd-2020-11](file:///C:\Users\maximiko\AppData\Local\Box\Box%20Edit\Documents\Bcz7kLGojEWIDH4iq4ToYQ==\10.5194\essd-2020-11)

Leontief, W. (1936). Quantitative input and output relations in the economic system of the United States. *The Review of Economic Statistics*, 18(3), 105-125. <https://doi.org/10.2307/1927837>

Leontief, W. (1970). Environmental repercussions and the economic structure: An input-output approach. *The Review of Economics and Statistics*, 52(3), 262-271. <https://doi.org/10.2307/1926294>

Miller, R. E., & Blair, P. D. (2009). *Input-output analysis: Foundations and extensions* (2nd ed.). Cambridge University Press. https://doi.org/10.1017/CBO9780511626982

United Nations. (2021). *Indicative Crop Classification for the Agricultural Census (ICC) Version 1.1*. Food and Agriculture Organization of the United Nations. <https://www.fao.org/3/cb7348en/cb7348en.pdf>
